# Diagnostic yield and safety of ultrasound-guided bowel mass biopsies in children

**DOI:** 10.1007/s00247-019-04472-8

**Published:** 2019-07-13

**Authors:** Kishore Minhas, Derek J. Roebuck, Alex Barnacle, Paolo De Coppi, Neil Sebire, Premal A. Patel

**Affiliations:** 1 Interventional Radiology, Radiology Department, Great Ormond Street Hospital for Children, Great Ormond Street, London, WC1N 3JH UK; 2 Department of Medical Imaging, Perth Children’s Hospital, University of Western Australia, Perth, Australia; 3 Department of Specialist Neonatal and Paediatric Surgery, Great Ormond Street Hospital for Children, London, UK; 4 Stem Cells and Regenerative Medicine Section, UCL Institute of Child Health, University College London, London, UK; 5 Wellcome/EPSRC Centre for Interventional & Surgical Sciences, University College London, London, UK; 6 Department of Pathology, Great Ormond Street Hospital for Children, London, UK

**Keywords:** Adolescents, Biopsy, Bowel, Children, Complication rate, Diagnostic yield, Lymphoma, Ultrasound

## Abstract

**Background:**

Traditionally, ultrasound (US)-guided bowel mass biopsies are avoided in favour of endoscopic or surgical biopsies. However, endoscopy cannot easily reach lesions between the duodenojejunal flexure and the terminal ileum and lesions not involving the mucosa may not be accessible via an endoscopic route.

**Objective:**

The aim of this study was to report our technique and to assess the diagnostic accuracy and safety of US-guided biopsy of bowel masses in children.

**Materials and methods:**

We conducted a 14-year retrospective review of US-guided bowel mass biopsies at a single paediatric hospital.

**Results:**

Twenty US-guided bowel mass biopsies were performed in 19 patients (median age: 6 years and 6 months, range: 22 months–17 years, median weight: 22 kg, range: 10.2–48.4 kg). For 14 biopsies, there was no other lesion that could potentially be biopsied. A percutaneous coaxial technique was used for 19 biopsies and a transanal non-coaxial biopsy was performed in 1. A median of 9 (range: 2–15) cores of tissue was obtained at each biopsy. The technical success rate and adequacy of diagnostic yield were 100%. The most common diagnosis was lymphoma, which occurred in 16 biopsies. Three biopsies contained mucosa. There was one complication out of 20 biopsies (5%, 95% confidence interval 0–15%): a self-limiting, post biopsy pyrexia. Nineteen procedures were accompanied by a bone marrow aspirate and/or trephine within 2 weeks of the bowel biopsy, only one of which was diagnostic.

**Conclusion:**

US-guided bowel mass biopsy can be performed safely in children, with a high diagnostic yield and low complication rate.

## Introduction

Traditionally, biopsies of masses arising from the bowel in children are performed endoscopically or surgically, via a laparotomy or laparoscopic approach.

The main limitation of performing an endoscopic biopsy of a bowel mass is that the reach of the endoscope is limited to the portions of the gastrointestinal tract between the mouth and the duodenojejunal flexure and between the rectum and the terminal ileum. Consequently, a bowel mass anywhere from the duodenojejunal flexure to the terminal ileum will be beyond the reach of an endoscopic biopsy. Furthermore, endoscopic biopsies are largely limited to the mucosa and submucosa and so intramural, subserosal or exophytic lesions may not be adequately sampled [1]. In such cases, endoscopic ultrasound (US)-guided biopsies may be considered. Endoscopic US, however, is also restricted by limited access to the small bowel beyond the duodenojejunal flexure. Surgical biopsies of bowel masses are not limited by location of the lesion but are invasive and have an associated morbidity [2–4].

A percutaneous US-guided approach is an alternative biopsy method. This technique has been described in adults and demonstrated to be both safe and effective [5] but has not previously been described in children. We hypothesise that US-guided biopsy of bowel masses may provide a valid alternative diagnostic approach for children in whom specimens suitable for histology cannot be easily obtained by endoscopy or other methods. The aim of this study was to report our technique and assess the diagnostic accuracy and safety of US-guided biopsy of bowel masses in children.

## Materials and methods

This single-centre retrospective study was exempted from institutional review board approval. Data were collected from a single paediatric hospital with a well-established interventional radiology programme. Patients were identified from a prospectively maintained interventional radiology database of biopsies. Inclusion criteria were all patients 0–18 years of age who underwent percutaneous or transanal US-guided biopsy of a bowel mass from June 2004 to January 2018. There were no exclusion criteria. Data sources included the radiology information system picture archiving and communication system, interventional radiology databases and electronic patient records.

Demographics recorded included patient age, weight at the time of biopsy and gender. Pre-procedural imaging findings, biopsy technique and histological result, post-procedural clinical course and imaging, and complications were recorded. Complications were graded according to the Society of Interventional Radiology classification of complications [6]. To assess whether a diagnosis could have been achieved by another method, the results of any bone marrow aspirates and trephines within a 2-week period and any endoscopic or surgical bowel biopsies within a month were recorded. Data were reviewed by two of the study investigators K.M., P.A.P who have 2 and 5 years of post-fellowship experience, respectively.

### Definitions and criteria

Technical success was defined as acquiring tissue from the bowel mass using a US-guided biopsy technique. The primary endpoint of clinical success was defined as an adequate diagnostic yield for a definitive histopathological diagnosis to be made. The secondary endpoints were complication rate, type and severity, categorised using Society of Interventional Radiology standards of practice committee classification of complications by outcome criteria [6]. A review of medical records up to discharge from the hospital following the commencement of chemotherapy or up to definitive surgical treatment was used to identify and categorise delayed complications.

Demographics, technical details and complications were analysed using descriptive statistics. Data are presented as median (range) unless otherwise stated. Further statistical comparison between subgroups was not performed due to the small sample size. Ninety-five percent confidence intervals (CI) were calculated for the proportion of complications.

### Bowel biopsy technique

**Fig. 1:**
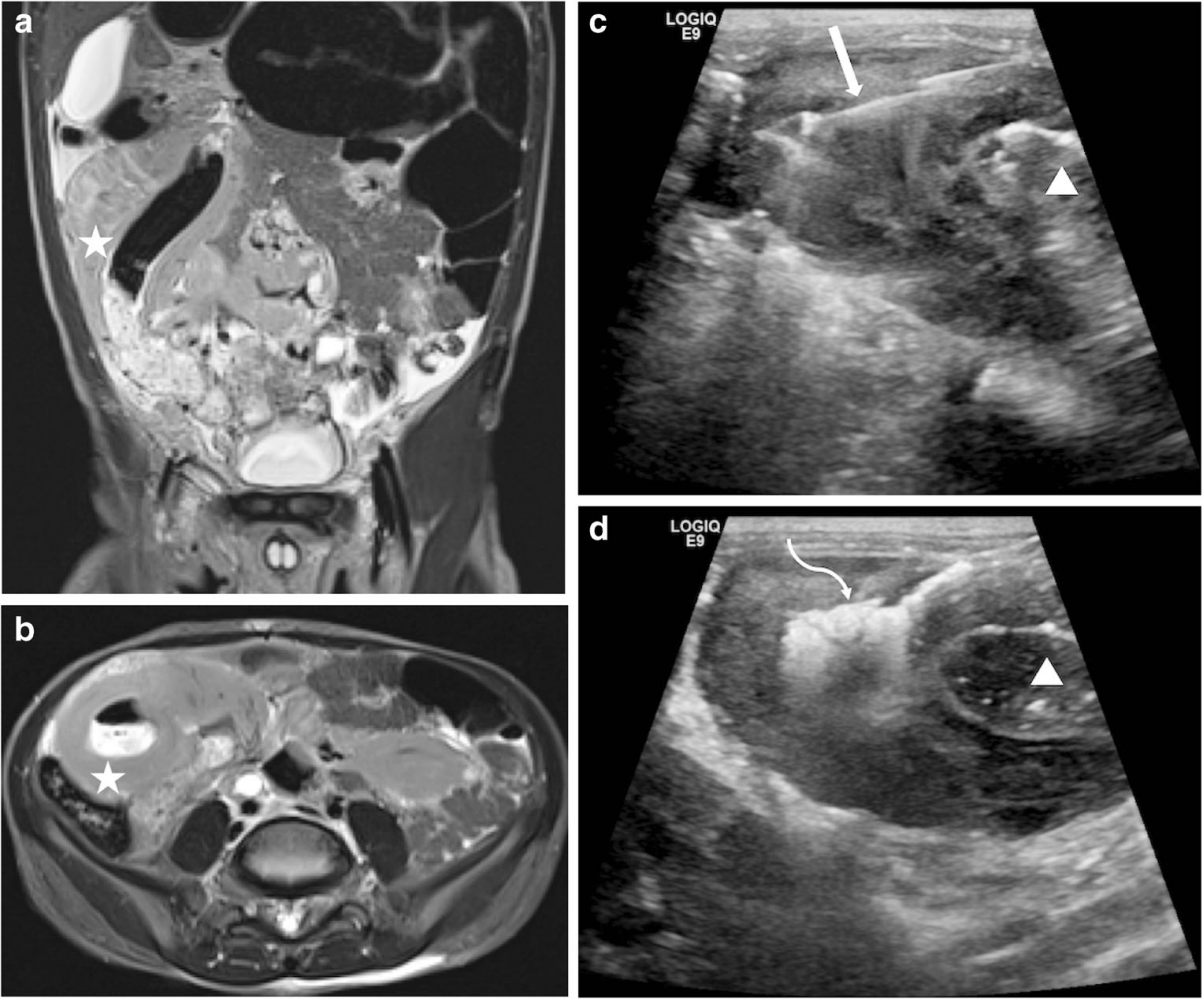
Transabdominal biopsy of a large bowel mass in a 3-year-old boy presenting with a scrotal mass and subsequently found to have synchronous bowel and testicular lesions. Coronal (**a**) and axial (**b**) pre-biopsy MRI demonstrate a bowel wall-based mass (*star*). Intra-procedural ultrasound (**c**) shows the biopsy needle in situ (*arrow*) adjacent to gas and fluid-filled lumen (*arrowhead*). Tract embolisation (**d**) with echogenic absorbable gelatin sponge (*arrowhead*). The histopathological diagnosis was Burkitt lymphoma

**Fig. 2:**
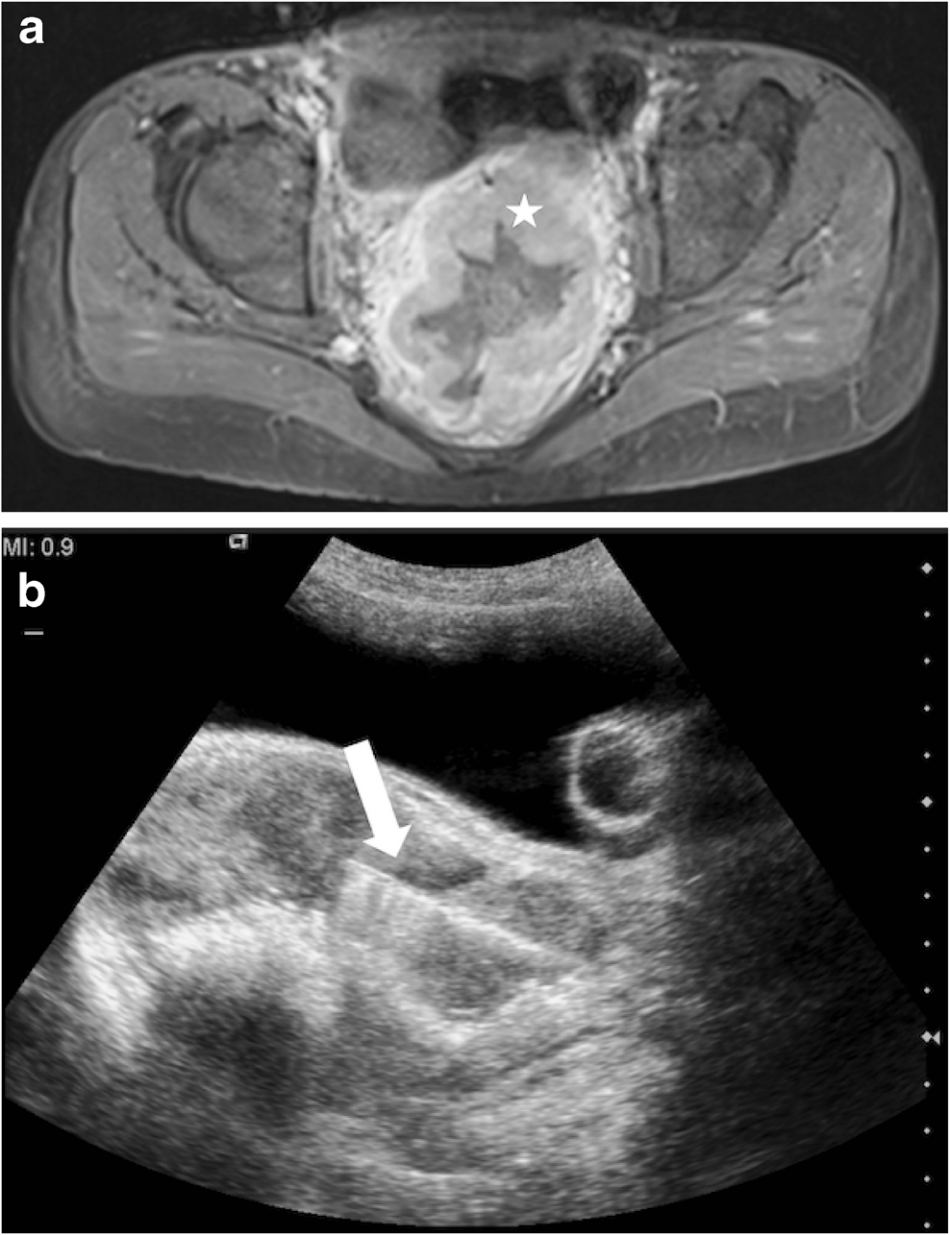
Transanal biopsy in 14-year-old girl presenting with a rising lactate dehydrogenase level following a heart transplant. Axial pre-biopsy MRI (**a**) demonstrates a grossly abnormal and thickened rectum (*star*). Intra-procedural ultrasound (**b**) using the urinary bladder filled with saline as an acoustic window (the urinary bladder catheter balloon can be seen in the bladder lumen) demonstrates the transanal biopsy route with a 14-gauge biopsy needle (*arrow*). The histopathological diagnosis was mature large B cell lymphoma

**Fig. 3:**
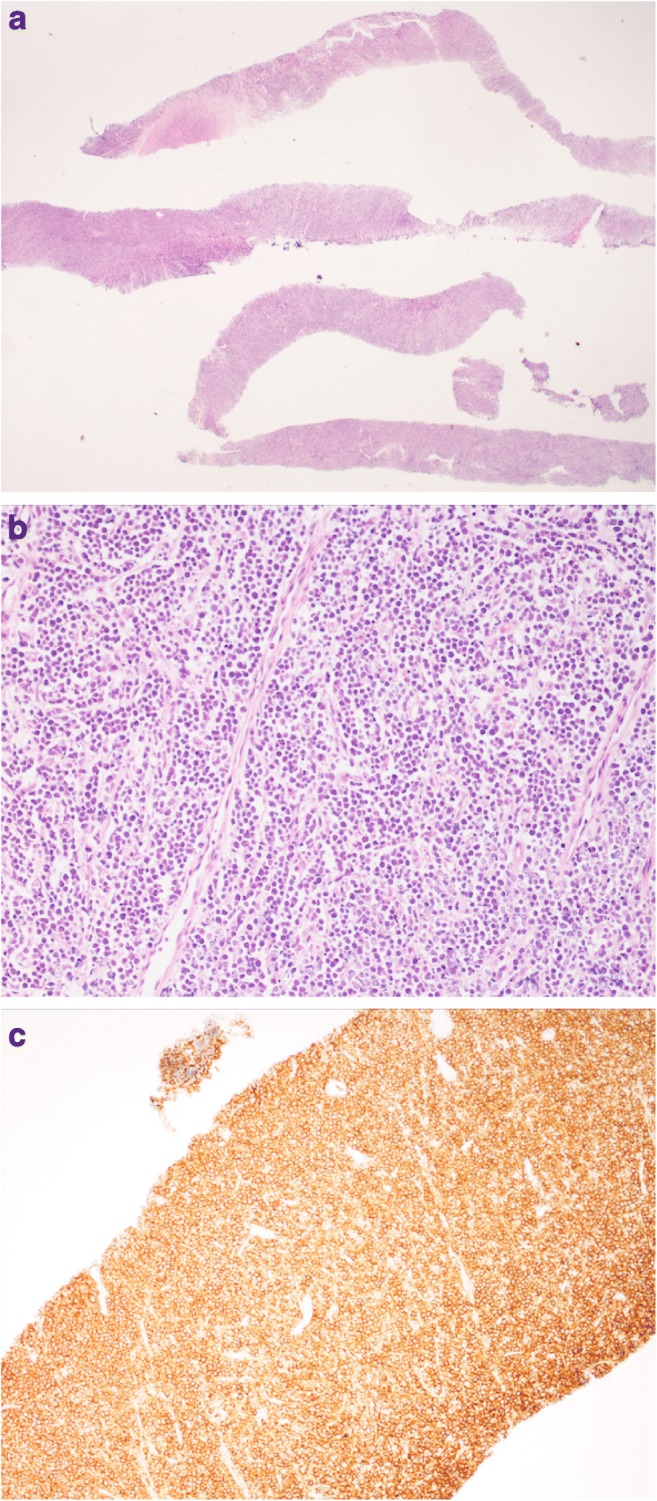
Photomicrographs of needle core biopsies from the patient in Fig. 1. **a** There is no normal underlying tissue but complete replacement by viable tumour composed of sheets of uniform tumour cells that infiltrated surrounding structures (haematoxylin and eosin stain: original magnifications × 20). **b** On high power, the tumour shows a uniform appearance of small ovoid cells, with focal Indian file morphology, with lines of cells between connective tissue structures (haematoxylin and eosin stain: original magnification × 200). **c** Tumour cells demonstrate diffuse strong membrane expression of CD20 confirming, B cell origin (DAB immunostain with haematoxylin counterstain; original magnification × 200)

The decision to perform a US-guided biopsy of a bowel mass was made in a multidisciplinary team meeting in conjunction with paediatric oncologists and paediatric surgeons, and after review of relevant prior imaging to ensure the presence of a suitable acoustic window. Pre-biopsy complete blood count and coagulation profile were reviewed and corrected following consultation with the haematology department. All biopsies were performed with the patient under general anaesthesia in an interventional radiology suite. Pre-procedure localisation of the bowel mass was performed with US (Logiq E9; GE Healthcare, Waukesha, WI) and the patient positioned accordingly. Depending on lesion accessibility, either a percutaneous transabdominal or transanal route was chosen. Transabdominal US guidance was used regardless of the approach to the lesion (Figs. 1 and 2). Biopsies were performed using a side-notch biopsy needle (Temno Evolution; CareFusion, San Diego, CA. If a coaxial system was used, the tract was plugged with compressed gelatin sponge pledgets (Gelfoam; Pfizer, New York, NY). Biopsy samples were delivered fresh for histopathological assessment (Fig. 3). No specific protocol was in place regarding the periprocedural administration of antibiotics; the decision to give antibiotics was at the discretion of the interventional radiologist.

## Results

### Patient population

Over a 14-year period, 20 US-guided bowel mass biopsies were performed in 19 children. Patient characteristics are given in Table 1. In 14 procedures, there was no other potential lesion available for biopsy. In the six procedures where there was a synchronous lesion that could have been biopsied, the multidisciplinary team agreed that the bowel mass was the safest to biopsy and/or most likely to give a diagnostic result. One patient underwent 2 biopsies 11 weeks apart due to the recurrence of the lesion. Of the 20 biopsies performed, the review of pre-procedural imaging suggested 16 were of lesions arising from the small bowel and 4 were of lesions arising from the large bowel.

**Table 1:** Patient characteristics

Patients, *n*	19
Biopsies, *n*	20
Age at biopsy, median (range)	6 years, 6 months (1 year, 10 months–17 years)
Weight at biopsy, kg, median (range)	22 (10.2–48.4)
Gender (male), *n* (%)	16 (84.2)
Diagnosis from each biopsy, *n* (%)	
Burkitt lymphoma	11 (55)
Diffuse large B cell lymphoma	5 (25)
Post-transplant lymphoproliferative disease	3 (15)
Necrotising granulomatous inflammation	1 (5)

### Technique

Nineteen of the 20 procedures were performed using a percutaneous transabdominal approach. A 15-gauge outer coaxial needle and 16-gauge biopsy needle were used for 14/19 (74%) biopsies and a 17-gauge outer coaxial needle and 18-gauge biopsy needle for 5/19 (26%). All percutaneous biopsies were followed by embolisation of the biopsy tract with compressed gelatin sponge pledgets (Figs. 1 and 3). One biopsy was performed using a transanal approach with a 14-gauge biopsy needle (Fig. 2) and no subsequent tract embolisation was performed. This biopsy was of a mass arising from the rectum, which could not be accessed percutaneously due to the overlying urinary bladder. The number of tissue cores obtained at each biopsy ranged from 2 to 15, with a median of 9. For 6/20 (30%) procedures, the child was already on a course of antibiotics for preexisting pyrexia at the time of biopsy. For the remaining 14 procedures, antibiotics were administered intra-procedurally in 1/14 (7%) procedures and not administered in 13/14 (93%) procedures.

### Outcomes

US-guided biopsy of the bowel mass was technically successful in all procedures. All biopsies yielded an adequate diagnostic yield to allow a histopathological diagnosis to be made (Fig. 3). The histological diagnoses are listed in Table 1. No repeat biopsies were required to obtain further immunohistochemical information. Three out of 20 (15%) biopsies performed included mucosa on histological examination inferring that there was mucosal breach in at least 3 biopsies.

Of the 19 patients who underwent US-guided bowel mass biopsy, 2 had undergone endoscopic biopsy less than 4 weeks before their radiologic biopsy. Histology from both endoscopic biopsies demonstrated focal active colitis only. On the subsequent US-guided biopsy, one of these lesions was diagnosed as diffuse large B cell lymphoma and the second as necrotising granulomatous inflammation. No patients had a surgical biopsy before or after the US-guided biopsy.

Bone marrow aspiration or trephine biopsy was performed within 14 days of 19/20 US-guided bowel mass biopsies. Only 1/19 (5%) showed marrow involvement by the disease process (Burkitt lymphoma); this diagnosis was consistent with the radiologic biopsy result. One out of 19 (5%) showed marrow infiltration by malignant cells but did not give a definite diagnosis, 5/19 (26%) showed reactive marrow changes only and 12/19 (64%) were normal.

### Complications

In the period of follow-up assessed (median: 45 days, range: 2–176 days), there was 1 complication out of 20 biopsies (5%, 95% CI: 0–15%). This was a self-limiting post-biopsy pyrexia that lasted for less than 24 h in a patient who was not on antibiotics before their biopsy and was not given intra-procedural antibiotics. This was the patient in whom a transanal biopsy was performed. There was no mucosa found on histological analysis of the sample. This corresponds to a minor A complication with no therapy required and no consequence, according to the Society of Interventional Radiology classification. Three other children were documented to have had pyrexia following their biopsy, but these children had been pyrexial before biopsy, and had been started on a course of antibiotics for this, so this was attributed to their underlying disease process. No patient developed a symptomatic or imaging-detected asymptomatic pneumoperitoneum and no patient required surgery to treat a complication.

## Discussion

Percutaneous image-guided bowel mass biopsy has been demonstrated to be both safe and effective in adults [5] but has not previously been described in children. Due to the small body size of children, transabdominal US can usually be performed using a high-frequency probe with excellent visualisation of the abdominopelvic structures including the small and large bowel. US provides dynamic information that cannot be obtained with other imaging techniques such as CT and MRI. It is minimally invasive, cost-effective, fast and does not require ionising radiation. US-guided biopsy is widely used in the diagnosis of other abdominopelvic lesions. It has high diagnostic accuracy, a low complication rate and low mortality [7].

Conventionally, biopsies of lesions arising from the gastrointestinal tract are obtained endoscopically. The endoluminal approach is limited to mucosal or submucosal lesions that arise between the mouth and the duodenojejunal flexure or between the terminal ileum and the rectum. Lesions between the duodenojejunal flexure and the terminal ileum are generally beyond the reach of an endoscope. Furthermore, lesions within reach of the endoscope that are intramural, subserosal or exophytic may be impossible to visualise and/or inadequately sampled. In these cases, one could attempt endoscopic US-guided fine-needle aspiration cytology or core biopsy. Endoscopic US-guided fine-needle aspiration has a high degree of sensitivity and specificity in adults [8]. Endoscopic US-guided core biopsy is, however, better when immunohistochemistry is needed, for example in submucosal tumours and lymphoma [8]. However, like conventional endoscopy, endoscopic US-guided biopsy is limited by poor access to the small bowel beyond the duodenojejunal flexure and proximal to the terminal ileum and is technically challenging. It has been shown to be uniformly poor when used in certain anatomical locations, such as the thickened gastrointestinal wall or focal intramural lesions [9, 10]. Traditionally, lesions that cannot be adequately biopsied endoscopically require a more invasive approach such as laparoscopic or open surgical biopsy. A surgical approach is not limited by the location of the lesion but is necessarily more invasive than percutaneous needle biopsy and may involve greater morbidity. Surgical biopsies of abdominal masses in children have a potential risk of intraoperative and postoperative haemorrhage, delay in initiating chemotherapy and other risks related to surgical procedures in general [2–4].

The US-guided approach is an alternative method for obtaining samples of bowel masses for histopathological evaluation. Previously reported sites of percutaneous fine-needle aspiration in adults include the stomach [1, 11, 12], small bowel [1, 13, 14] colon [1, 14, 15] and rectum [1]. Percutaneous image-guided fine-needle aspiration of gastrointestinal lesions has been reported to be safe in adults [1, 14]. However, in a series of 20 adults there were 5 negative samples (showing benign colonic epithelium, benign stroma, acute inflammation or benign lymphoid cells) [1].

We have demonstrated that US-guided biopsy of bowel masses can be performed safely and successfully in children. The location of the lesion does not appear to preclude a US-guided approach. Only one mass, arising from the rectum, was thought to be unsafe to biopsy using a transabdominal approach due to the overlying urinary bladder, but it was successfully biopsied using a transanal approach and transabdominal US guidance.

In the vast majority of these cases, the underlying pathological process is essentially homogeneous diffuse disease, such as lymphoma and post-transplant lymphoproliferative disease, and the precise site of biopsy is of no particular relevance for establishing or staging the disease. Due to the homogenous nature and diagnostic immunohistochemical profile of most of these disease processes, provided at least some viable tissue is present, the amount of material required is very small. This is in contrast to a biopsy of a lymph node, for example, where there may be only focal involvement; here, the amount of material and specific site of biopsy are of much greater relevance [16]. In this series, masses arose from the small and large bowel. A definitive clinical diagnosis was made from all biopsies in this series.

There was only one minor (Society of Interventional Radiology category A) complication of a self-limiting, post-biopsy pyrexia suggesting that the procedure is safe. This case of pyrexia followed the transanal biopsy when pre- or intra-procedural prophylactic antibiotics were not administered. There were at least three percutaneous biopsies in which the bowel mucosa was breached without any complication. These mucosal breaches were non-intentional, and therefore this procedure should be considered “clean contaminated” according to the Society of Interventional Radiology Standards of Practice [17]. Given that bowel mass biopsies can be considered a clean contaminated procedure, and that patients may become immunocompromised with subsequent treatment, an argument could be made for the use of prophylactic antibiotics. Against this, the low frequency of complications in children who did not receive pre- or intra-procedural antibiotics would suggest that the need for antibiotic prophylaxis should be based on an individual patient’s risk factors.

All patients in our study population were inpatients, but given the absence of any major complications it is possible that patients could undergo this procedure during a single-day admission.

All procedures in this series yielded a definitive diagnosis and all but one proved to be an aggressive haematological process (lymphoma or post-transplant lymphoproliferative disease). All of these patients were treated medically and did not require surgery, and so US-guided biopsy avoided laparotomy or laparoscopy. Only one patient in the series, in whom the biopsy diagnosed a necrotising granulomatous inflammatory lesion, went on to have an elective laparotomy and left hemicolectomy 8 days after biopsy for surgical resection of this mass.

Of the 20 cases in this series, 2 had a prior endoscopic biopsy showing only inflammatory changes of colitis. In these two patients, the subsequent US-guided biopsy showed diffuse large B cell lymphoma and a necrotising granulomatous inflammatory lesion. If US-guided biopsy had not been performed in these patients it would have delayed their diagnosis and management and possibly increased their morbidity.

Bone marrow aspirate has routinely been performed as part of lymphoma staging. However, patients with early-stage disease rarely have bone marrow involvement [18]. In this series, only 1/19 bone marrow biopsies was diagnostic (Burkitt lymphoma) and this was consistent with the diagnosis made on the US-guided biopsy. The remaining 18/19 bone marrow aspirates were nondiagnostic. This shows that the diagnostic yield from bone marrow aspirate and/or trephine biopsy is inferior to US-guided biopsy of the bowel mass, most likely because there is not involvement of the marrow by the disease process. In these cases, without performance of a biopsy of the bowel mass, a diagnosis would not have been achieved.

This study has several limitations. It is a retrospective review of patients in whom a biopsy was attempted. Patients for whom a US-guided biopsy of a bowel wall mass was requested but not performed after review of available diagnostic imaging (CT, MRI and US) because it was thought to be not technically feasible, and were not included. Therefore, no attempt to assess the proportion of bowel masses that may be suitable for this technique can be made. The nature and location of lesions biopsied were not recorded such that useful data regarding lesion location that may be suitable for US-guided biopsy could be obtained. If conducting a prospective assessment, this information would be useful. There were no cases in which the core biopsies obtained were inadequate for evaluation or in which significant complications arose. However, the number of patients is small.

## Conclusion

This study demonstrates that percutaneous or transanal US-guided bowel mass biopsies in children are technically feasible. The diagnostic yield in this study was 100% and minor complications occurred in 5%. The procedure avoids the use of more invasive techniques or ionising radiation. The use of prophylactic antibiotics remains controversial. This technique could be considered the first option for diagnosing bowel masses that can be visualised on US, or as an alternative when an endoluminal approach is not possible or has not provided definitive results.
